# Microstructure and properties of additively manufactured Al–Ce–Mg alloys

**DOI:** 10.1038/s41598-021-86370-4

**Published:** 2021-03-26

**Authors:** K. Sisco, A. Plotkowski, Y. Yang, D. Leonard, B. Stump, P. Nandwana, R. R. Dehoff, S. S. Babu

**Affiliations:** 1 Material Science and Engineering, University of Tennessee-Knoxville, Knoxville, TN USA; 2 Materials Science and Technology Division, Oak Ridge National Laboratory, Oak Ridge, TN USA; 3 Manufacturing Science Division, Oak Ridge National Laboratory, Oak Ridge, TN USA; 4 Mechanical, Aerospace and Biomedical Engineering, University of Tennessee-Knoxville, Knoxville, TN USA

**Keywords:** Materials science, Structural materials

## Abstract

Additive manufacturing of aluminum alloys is largely dominated by a near-eutectic Al-Si compositions, which are highly weldable, but have mechanical properties that are not competitive with conventional wrought Al alloys. In addition, there is a need for new Al alloys with improved high temperature properties and thermal stability for applications in the automotive and aerospace fields. In this work, we considered laser powder bed fusion additive manufacturing of two alloys in the Al–Ce–Mg system, designed as near-eutectic (Al–11Ce–7Mg) and hyper-eutectic (Al–15Ce–9Mg) compositions with respect to the binary L → Al + Al_11_Ce eutectic reaction. The addition of magnesium is used to promote solid solution strengthening. A custom laser scan pattern was used to reduce the formation of keyhole porosity, which was caused by excessive vaporization due to the high vapor pressure of magnesium. The microstructure and tensile mechanical properties of the alloys were characterized in the as-fabricated condition and following hot isostatic pressing. The two alloys exhibit significant variations in solidification structure morphology. These variations in non-equilibrium solidification structure were rationalized using a combination of thermodynamic and thermal modeling. Both alloys showed higher yield strength than AM Al-10Si-Mg for temperatures up to 350 °C and better strength retention at elevated temperatures than additively manufactured Scalmaloy.

## Introduction

Additive manufacturing (AM) allows for geometric flexibility in part production and offers an increased design space, enabling complex cooling channels, mesh geometries, and sophisticated near net shape parts that are impossible to produce with conventional manufacturing techniques^1^. Specifically, in aluminum alloys, the use of AM could allow for the light-weighting of structural components in aerospace and automotive applications. However, conventional high-strength wrought aluminum alloys are poorly suited for the complex thermal cycles found in AM^2^ due to their propensity for solidification cracking^3^. For example, AM of alloy compositions similar to 7075^4^ and 2024^5^ showed significant processing limitations due to solidification cracking. While solidification cracking can be mitigated through careful processing parameter design in simple parts (*e.g.*, cubes) optimized parameters do not necessarily translate to complex parts.

The difficulties in processing of traditional alloys has led the aluminum additive community to widely adopt near-eutectic Al-Si, more specifically the Al–10Si–Mg alloy^6–13^. These alloys exhibit excellent castability and resistance to solidification cracking, but show much lower strength than conventional wrought alloys, and poor strength retention at elevated temperatures^14–16^. The rapid solidification rates of AM results in higher yield strength compared to conventional processing of similar compositions^15,17^. However, this improvement in strength has been attributed to super-saturation of Si in the Al matrix, and the increase in strength quickly dissipates due to Si precipitation at elevated temperatures.

**Figure 1:**
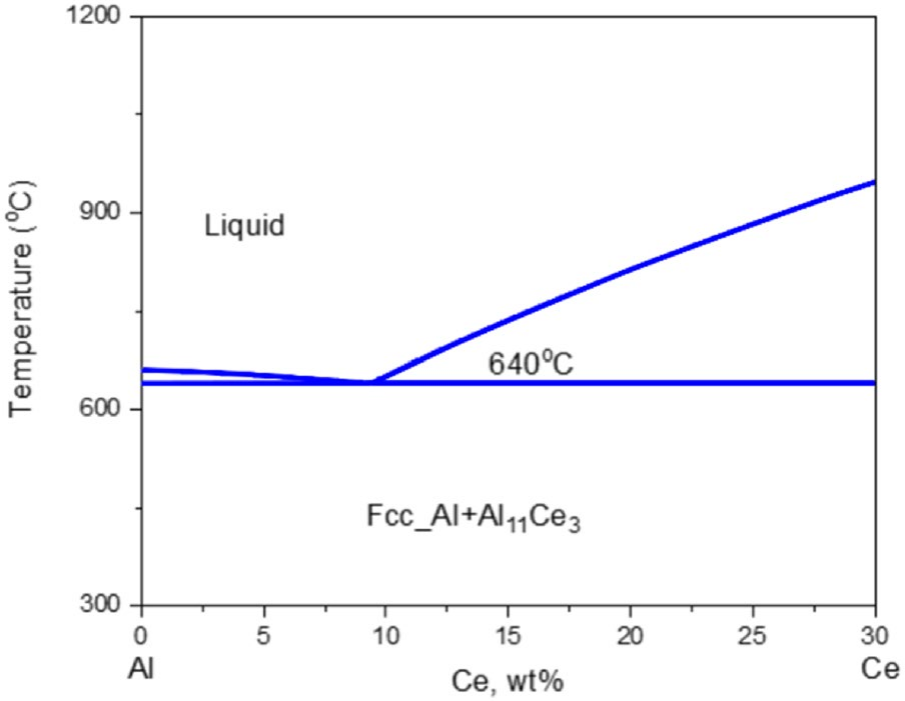
Al-Ce Binary Phase Diagram from 0 to 30 (wt%).

These challenges in AM processing of conventional wrought Al alloys, and the limited performance of Al–Si alloys, has prompted the examination of new Al alloys specifically designed for AM^18^. Among these, the Al–Ce system^19^ is particularly interesting due to its thermal stability and resistance to solidification cracking in castings^20,21^. The binary Al–Ce system exhibits a eutectic reaction at approximately 10 wt% Ce between Al and the ${\mathrm{Al}}_{11}{\mathrm{Ce}}_{3}$ intermetallic phase, and near-eutectic compositions result in excellent castability. The phase diagram shown in Fig. 1, shows the Al-Ce binary phase diagram from 0 to 30 wt%.

The cast microstructure of the Al-Ce binary system has been shown to be thermally stable to temperatures up to 500 $^\circ \mathrm{C}$ tested to 3024 h^22^, likely due to the limited solubility of Ce in the FCC-Al matrix, which slows the kinetics for Ostwald ripening^23^, and also exhibits promising creep properties^24^. The high solidification rates characteristic of additive manufacturing have been shown to significantly refine the microstructure of these alloys, resulting in an increase in hardness compared to cast structures^25–27^. However, the strength of these alloys is derived primarily from dispersion strengthening from the Al_11_Ce_3_ intermetallic particles, while the Al matrix is comparatively soft. As a result, there is a significant design space for exploring additional alloying elements. For example, Manca et al. successfully demonstrated additive manufacturing of an Al-Ce-Cu alloy with yield strength up to 275 MPa and ultimate tensile strength up to 460 MPa with good thermal stability^28^. In addition to Manca et al. multiple authors are investigating Scallmalloy type alloys where an alloy base of Al–Mg can be modified with Sc and Zr, in some cases a high percentages of Sc is used in order to produce parts^29,30^.

The purpose of this work is to investigate the Al–Ce–Mg ternary system as a viable candidate for printable Al alloys. The high solubility of Mg in the Al matrix is attractive for adding solid solution strengthening, and Al–Ce–Mg cast alloys have shown a significant increase in hardness and excellent thermal stability compared to binary Al-Ce alloys^31,32^. In this study, we investigate AM of two Al–Ce–Mg alloys, one near-eutectic and one hypereutectic with respect to the $\mathrm{L}\to \mathrm{Al}+{\mathrm{Al}}_{11}{\mathrm{Ce}}_{3}$ reaction, and both with significant additions of Mg. This work describes the processing of these two alloys via AM and resulting microstructures and mechanical properties as a function of temperature. The thermal stability of the alloy is assessed following hot-isostatic pressing, and the variation in microstructure and properties is rationalized by considering variation in the thermal characteristics of the AM process and the alloy thermodynamics and kinetics under highly non-equilibrium cooling conditions.

## Experimental procedure

### Additive manufacturing and materials

Two Al–Ce–Mg alloys were designed for additive manufacturing: Al–11Ce–7Mg and Al–15Ce–9Mg, with composition given in wt%. With respect to the $\mathrm{L}\to \mathrm{Al}+{\mathrm{Al}}_{11}{\mathrm{Ce}}_{3}$ binary eutectic reaction, the first alloy is a near-eutectic composition, while the second is hypereutectic. Mg was added to act as a solid solution strengthener, as it has among the highest solubility of any element in the FCC Al matrix. Ingots of the targeted compositions were produced and then nitrogen gas atomized. Powder was then sieved for average particle size distributions between 20 and 63 µm.

**Figure 2:**
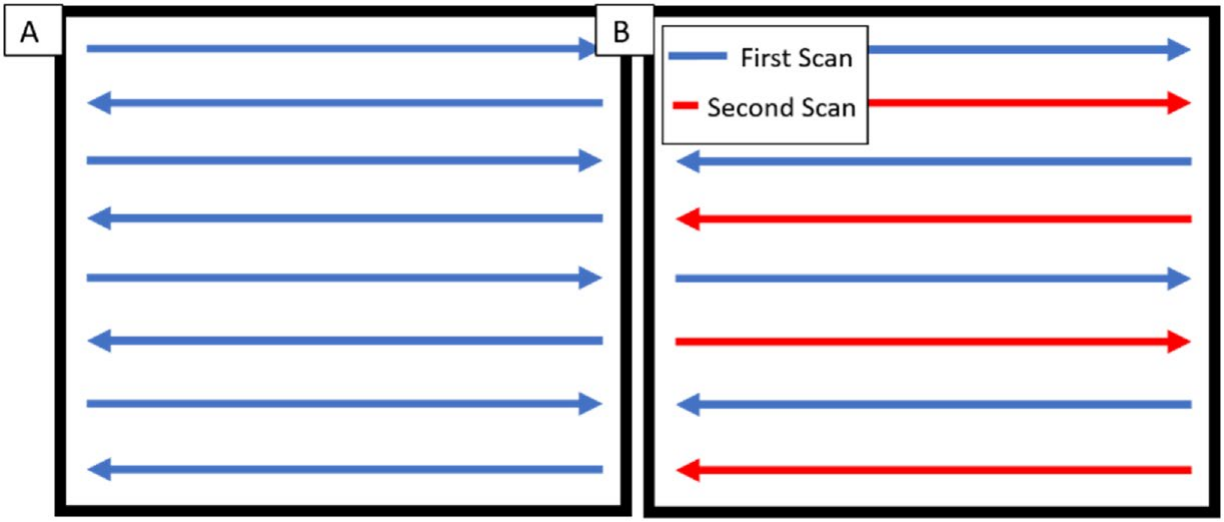
Comparison of (**A**) Conventional Raster and (**B**) Skip raster techniques.

Additive manufacturing was performed using a Concept Laser M2 laser powder bed fusion system. A design of experiments was performed on each alloy to determine optimal process conditions which were then used to produce tensile bars. Two different scan patterns were used for the hypereutectic alloy. The first being a conventional raster pattern, and the second a skip raster, which was developed to reduce the heat input into localized regions. The skip raster strategy follows the same general principal of a traditional raster strategy, but every hatch spacing is doubled. After the first scan section is complete across a part, the second scan comes back and fills in the previously un-melted regions. The delay in re-melting allows for local temperature to drop which appears to have a profound effect on reducing the amount of keyhole porosity in the part. Figure 2 shows a schematic comparison of the skip raster strategy to a conventional raster pattern. Hot Isostatic press (HIP) processing was used on both alloys, a low temperature HIP developed for other alloys contain similar processing conditions to the current work^33^. The chemical analysis of the various states of processing were determined using inductively coupled plasma. A summary of the composition in each condition is shown in Table 1.

**Table 1:** Measured Chemical Composition of Atomized Powder and As-Fabricated Parts. All values are in wt%.

Sample	Al	Ce	Mg	Si	Cu	Fe	O
Near Eutectic Powder	80.75	10.91	7.54	0.22	0.01	0.07	.125
Near Eutectic AM	81.72	11.1	6.45	0.24	0.01	0.07	0.035
Hypereutectic Powder	75.67	14.50	9.22	0.24	0.01	0.08	.0057
Hypereutectic AM (Conventional Raster)	77.28	14.53	7.44	0.39	0.01	0.08	< .0005%
Hypereutectic AM (Skip Raster)	76.36	14.45	8.45	0.37	0.01	0.08	< .0005%

### Mechanical testing

Blank cylinders of approximately 15 mm diameter and 105 mm length were machined into tensile bars in accordance with the ASTM E8 standard^34^ with a 6.35 mm gage diameter. Tensile testing was performed using a strain rate of $5\times {10}^{-4}{\mathrm{s}}^{-1}$ for both room temperature and elevated temperature testing. Elevated temperature testing used a temperature ramp rate of 10 $^\circ$ C/min and a soak time of 30 min to ensure equal heating across the specimen.

### X-ray diffraction

X-Ray Diffraction (XRD) was collected using a PANalytical Empyrean instrument configured with a Bragg–Brentano geometry. Cu–K$\mathrm{\alpha }$ radiation was used (45 kV and 40 mA). Incident and diffracted beam optics include programmable divergent slits, anti-scattering slits and a PIXcel detector. Data was collected between 10° and 120° $2\theta$, with a step size of 0.026°. Phase Identification was performed with the Inorganic Crystal Structure Database (ICSD)^35^.

### Microscopy

Optical, Scanning Electron Microscopy and Scanning Transmission electron microscopy (STEM) high angle annular dark field (HAADF) images were collected. The Optical microscopy was acquired on a Zeiss Axio Imager. The SEM was acquired on a Zeiss Evo. Focused Ion Beam Milling (FIB-M) was preformed using a Hitachi NB5000 FIB/SEM instrument. STEM images were collected using an FEI Talos F200X, using a symmetric A-TWIN objective lens integrated with SuperX EDS system.

### Hardness testing

Samples were polished to a surface finish of $0.5\mathrm{ \mu m}$ using a diamond paste before Vickers indentation at room temperature was performed on a LECO 55 Automatic Hardness tester. Using a 1 kg load, the indenter was kept in contact with the surface for 10 s. Thirty-six indentations were taken for each sample and the average hardness was calculated.

### CALPHAD modeling

Computer coupling of phase diagrams and thermo-chemistry, *i.e.*, the CALPHAD approach^36^, was used to aid understanding of the as-solidified microstructure. In this approach, the Gibbs energy of individual phases was modeled based on crystal structure and phase chemistry. The model parameters were obtained through an optimization procedure that aims at consistently reproducing the experimentally assessed phase equilibria and thermodynamic properties by the model-calculated ones. The thermodynamic database, *i.e.*, a compilation of Gibbs energy functions of individual phases, was modeled in sequence from unary, binary, and ternary. The Gibbs energy functions of the three unary systems Al, Ce and Mg were adopted from the SGTE (Scientific Group Thermodata Europe) database compiled by Dinsdale^37^. The Gibbs energy functions of phases in the Al–Ce–Mg system were adopted from previous work done by Gröbner et al.^38^. The compiled thermodynamic database was then coupled with Pandat software^39^ to calculate liquidus projection and solidification paths.

### Solidification condition calculations

To understand the influence of process conditions on microstructure development, a simplified semi-analytical heat conduction model was utilized to approximate the trends in solidification conditions. Similar approaches have been successfully implemented in other studies to rationalize the influence of process conditions on microstructure and defects^40–44^. The model used here relies on the mathematical solution for a moving volumetric Gaussian heat source originally derived by Nguyen et al.^45^, and uses an adaptive Gaussian quadrature scheme to efficiently and accurately compute the melt pool behavior over long length and time scales^46^. The model calculates both the thermal gradient and solid–liquid interface velocity at the solidification front, which was taken here to occur at the eutectic temperature (see “Solidification structure” section for discussion). To capture the solidification conditions throughout the bulk of the material, multiple simulations were run to represent at least 5 layers (250 µm) of representative solidified material. Additionally, to ensure a high resolution (2.5 µm) without generating an infeasibly large amount of data, the domain was set to be a cylinder of radius 1 mm located at the center of the full cylinder. This assumption does not invoke any numerical inaccuracies for the simulations, since the analytic solution for temperature at a point is spatially independent of nearby points. The thermophysical properties of the Al–Ce–Mg alloys were approximated as being equivalent to A356 with the values being taken from Overfelt et al.^47^ at around ${T}_{eut}$ (Table 2).

**Table 2:** Simulation parameters.

Properties	Value	Units
**A356:**		
Density,$\rho$	2500	$\mathrm{kg}/{\mathrm{m}}^{3}$
Specific heat capacity,${c}_{p}$	$1080$	$\mathrm{J}/\left(\mathrm{kg K}\right)$
Thermal conductivity,$k$	$190.0$	$\mathrm{J}/\left(\mathrm{m s K}\right)$
Eutectic Temperature,${T}_{eut}$	$723$	K
Absorption Efficiency,$\eta$	35%	–

## Results

### Porosity characterization

**Figure 3:**
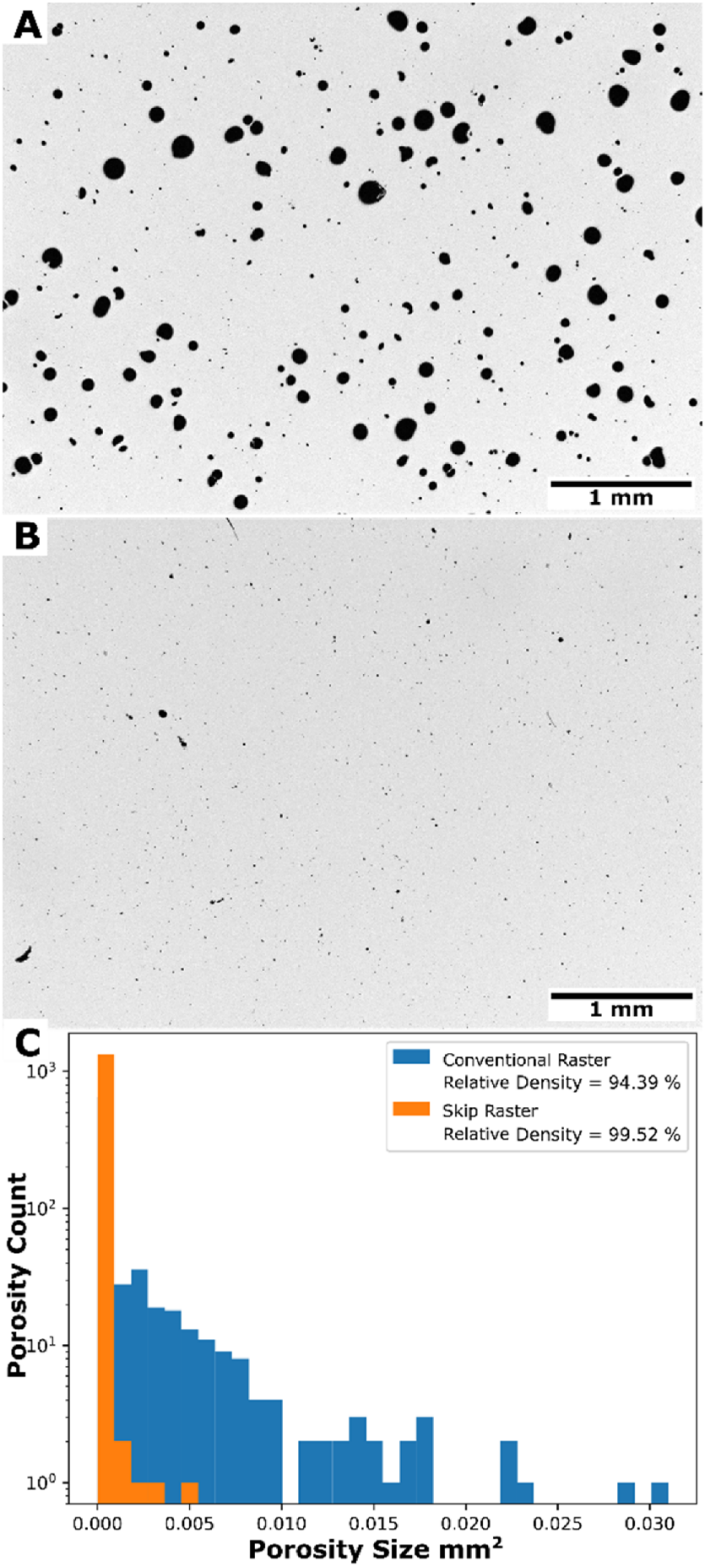
Comparison on Conventional (**A**) and Skip raster (**B**) for the hypereutectic alloy. With (**C**) being a comparison of the two relative densities.

Development of the skip raster pattern shown in Fig. 2 was motivated by a significant amount of porosity observed for conventional raster patterns in the hyper-eutectic alloy. Figure 3 shows a comparison of optical micrographs showing porosity distributions for the convention raster pattern and the skip raster pattern in the hypereutectic alloy. The conventional raster pattern contained a relative density of 94.39% and the skip raster contain a relative density of 99.52%. The size and morphology of pores for the conventional raster pattern is consistent with keyhole porosity^48^. Based on these results, the skip raster was used for production of tensile coupons with the hyper-eutectic alloy. Thee skip raster condition will be used as a basis of comparison for the remainder of this work.

### Microstructure characterization

**Figure 4:**
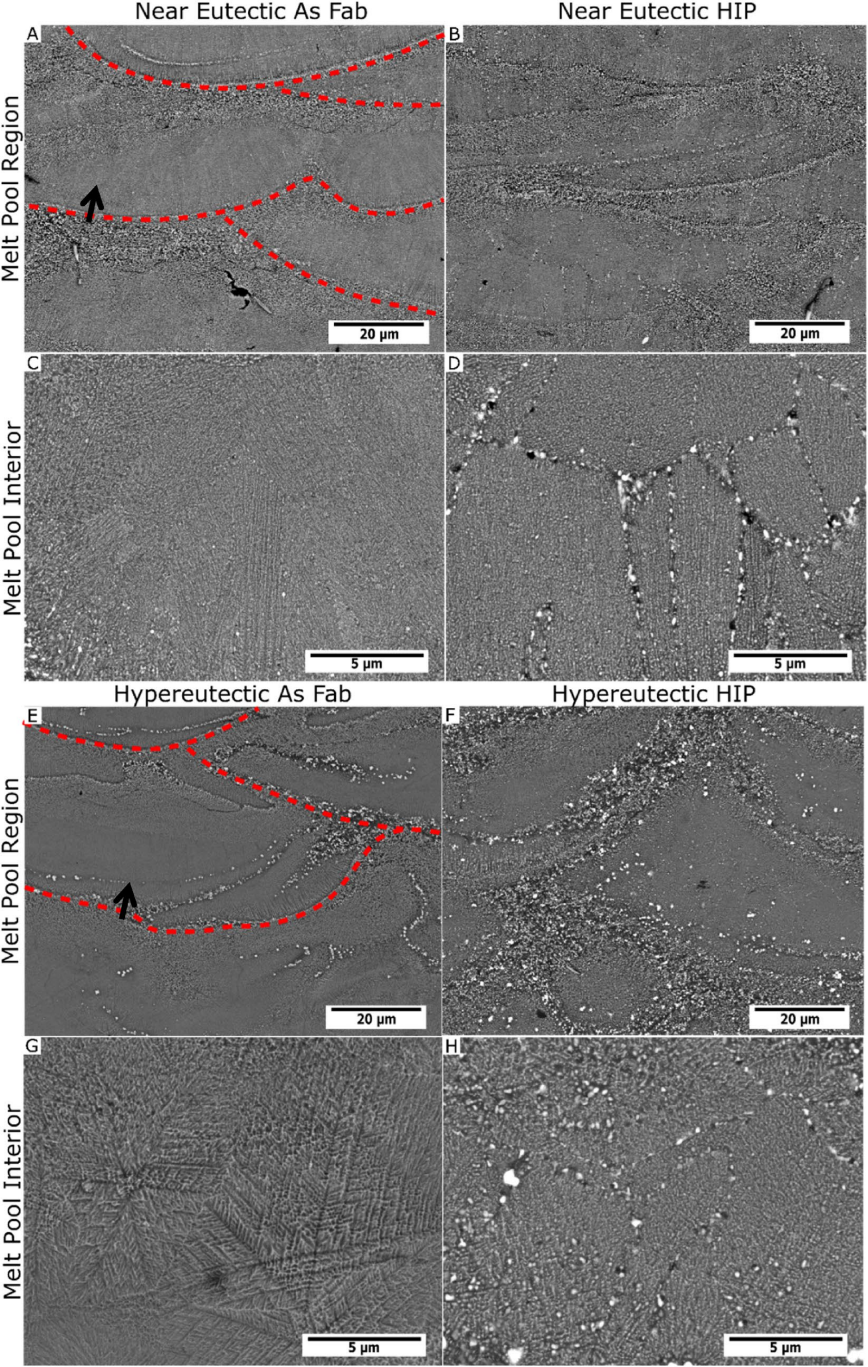
SEM of Selected Regions of Near Eutectic and Hypereutectic before and after HIP. Where (**A**) and (**C**) are the as fabricated Near Eutectic, (**B**) and (**D**) are the HIP Near Eutectic samples, (**E**) and (**G**) are the as fabricated Hypereutectic samples, and (**F**) and (**H**) are the HIP Hypereutectic samples.

SEM micrographs of the as-fabricated and post-HIP microstructures for each alloy are summarized in Fig. 4. Both alloys exhibit heterogeneous microstructural distributions that appear to correspond with the melt pool shape. The micrographs show similar trends in both alloys in which a coarser phase distribution is observed at the edge of the melt pools, which are indicated with red dashed lines. Away from the edge of the melt pool, a transition to a finer region occurs. In the HIP samples, ${\mathrm{Al}}_{11}{\mathrm{Ce}}_{3}$ coarsens preferentially on the grain boundaries. The growth of the ${\mathrm{Al}}_{11}{\mathrm{Ce}}_{3}$ phase happens in both alloys, but the grains of the hyper-eutectic samples are less defined.

**Figure 5:**
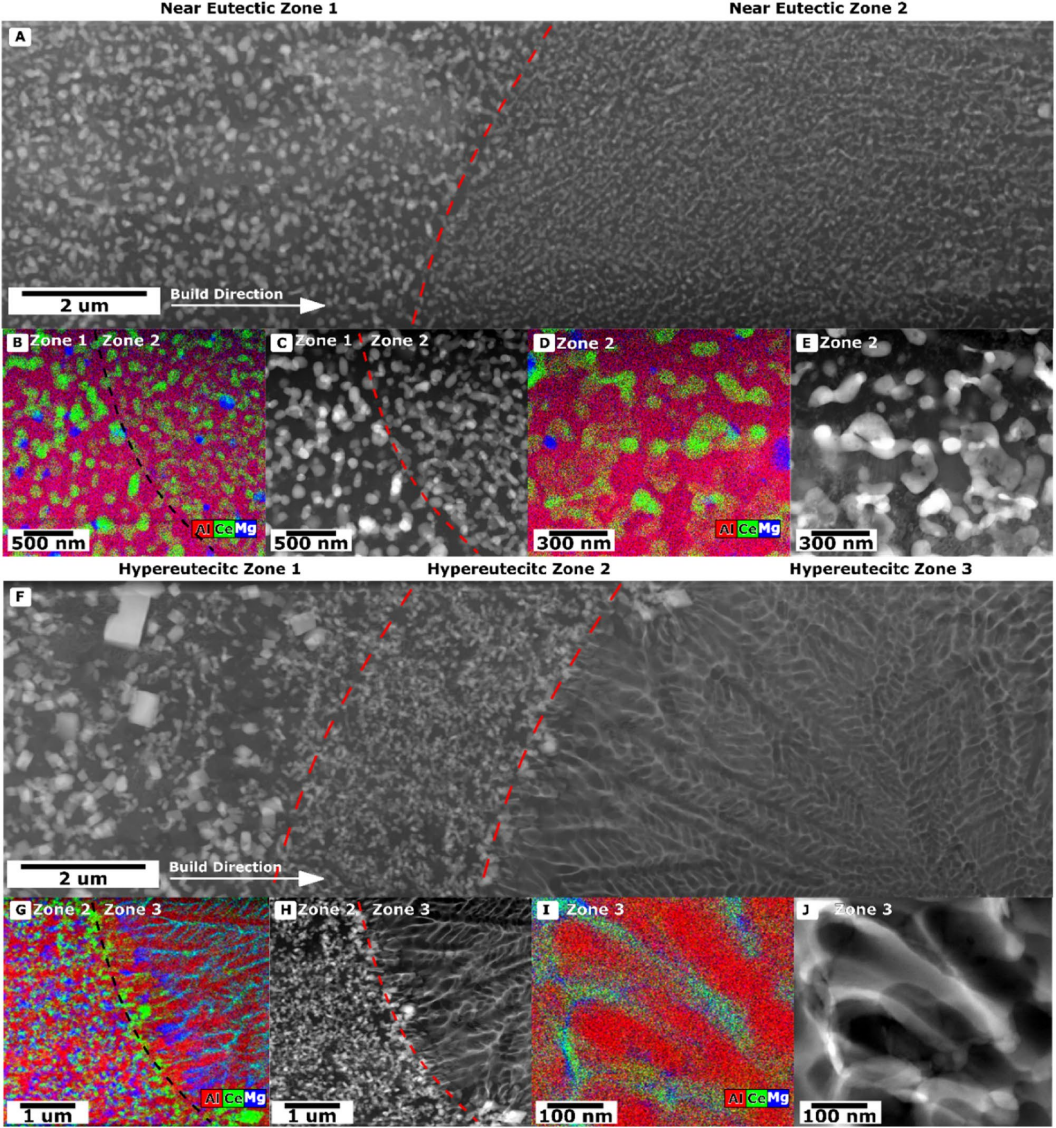
(**A**) HAADF View of Near Eutectic TEM Foil across the weld pool boundary. A symbolic arrow is shown in Fig. 4A. (**B**) STEM Combined Map of Elements for Region C for the Near Eutectic Alloy. (**C**) Bright Field of Dendritic region across Zone 1 and Zone 2 for the Near Eutectic Alloy. (**D**) STEM Combined Map of Elements for E for the Near Eutectic Alloy. (**E**) HAADF View across the edge of the weld pool into the dendrite region for the Near Eutectic Alloy. (**F**) HAADF View of TEM Foil for Hypereutectic Alloy across the weld pool boundary. A symbolic arrow is shown in figure. (**G**) STEM Combined Map of Elements for Region H for the Hypereutectic Alloy. (**H**) Bright Field of Dendritic region across Zone 2 and Zone 3 for the Hypereutectic Alloy (**I**) STEM Combined Map of Elements for J for the Hypereutectic Alloy. (**J**) HAADF View across the edge of the weld pool into the dendrite region for the Hypereutectic Alloy.

For closer observation of the alloy microstructures, STEM micrographs and STEM-EDS maps were taken from as-fabricated samples of both alloys (Fig. 5). In general both alloy lift outs were focused on the external of the weld pool moving inwards toward the center of the weld pool. Two distinct regions are present in the near-eutectic alloy, the first being a region containing globular ${\mathrm{Al}}_{11}{\mathrm{Ce}}_{3}$ particles surrounded by Al (labeled Zone 1), and the second (Zone 2) appears to be a fibrous eutectic, similar to what is sometimes seen in Al-Si alloys^49–51^. The hyper-eutectic alloy (Fig. 5F) exhibits three distinct regions (denoted Zones 1, 2, and 3). Zone 1 from the hypereutectic alloy appears to contain larger blocky ${\mathrm{Al}}_{11}{\mathrm{Ce}}_{3}$ particles that could indicate primary solidification. Zone 2 contains fine globular ${\mathrm{Al}}_{11}{\mathrm{Ce}}_{3}$ particles. Zone 3 contains Al dendrites and ${\mathrm{Al}}_{11}{\mathrm{Ce}}_{3}$ as a secondary phase.

The STEM-EDS maps show the expected Ce-rich Al_11_Ce_3_ intermetallic and Al matrix. However, both alloys also exhibit an additional Mg-rich intermetallic that is finely distributed within the microstructure, generally below 100 nm in size. Additionally, at the border across Zone 2 and into Zone 3 in the hypereutectic alloy, there is apparent segregation of Ce and Mg, with Ce enriching the boundary between the two zones and significant Mg enrichment in the interdendritic region at the edge of Zone 3.

### X-ray diffraction data analysis

**Figure 6:**
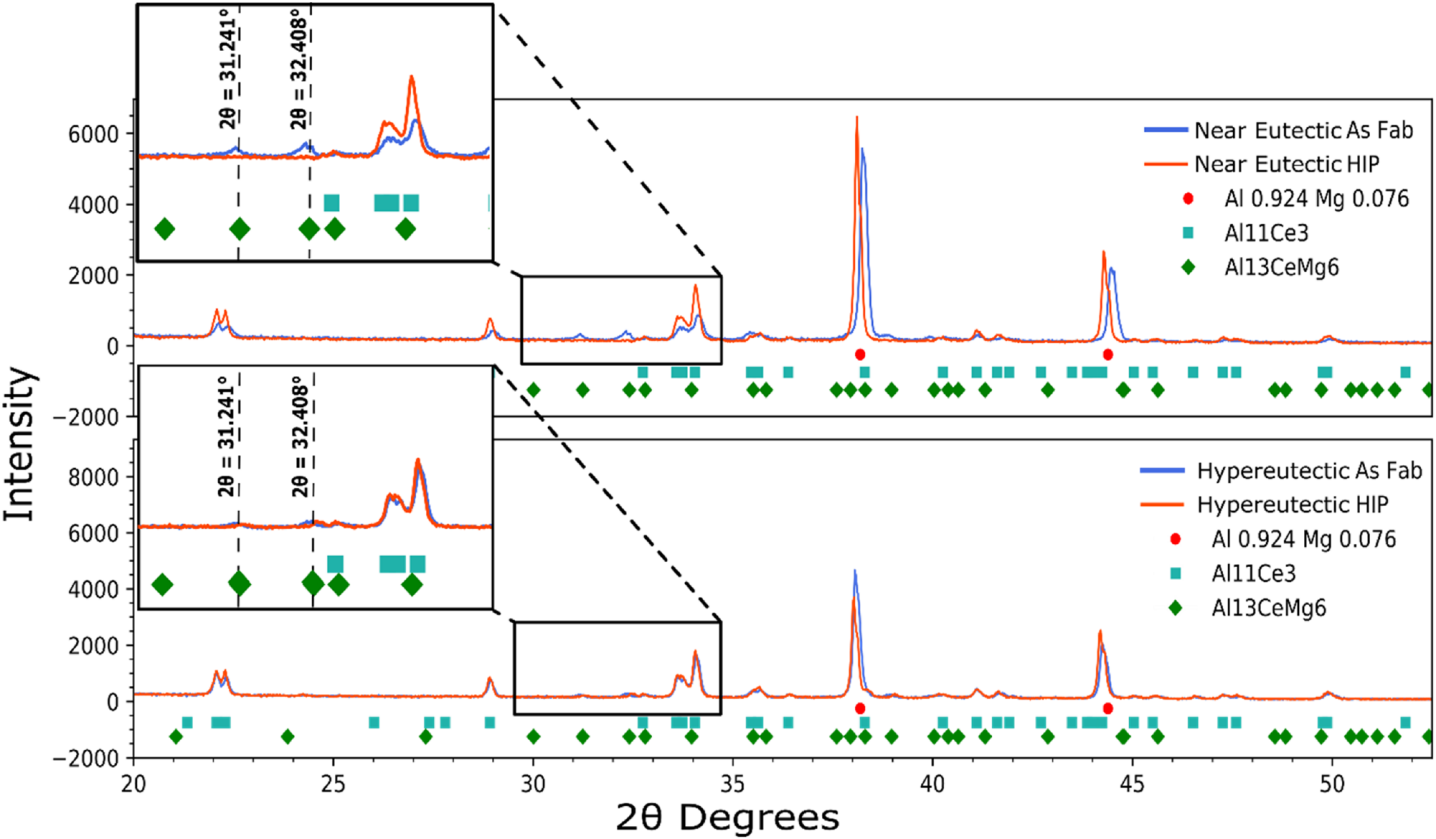
X-ray Diffraction Data form the Near Eutectic and Hypereutectic. The figure includes expected phases from the Scheil solidification diagrams, excluding the ${\mathrm{AlMg}}_{\upbeta }$ phase.

XRD spectra were collected for both alloys in the as-fabricated and HIP conditions as shown in Fig. 6. The XRD spectra are consistent with three phases: FCC Al, ${\mathrm{Al}}_{11}{\mathrm{Ce}}_{3}$ and ${\mathrm{Al}}_{13}{\mathrm{CeMg}}_{6}$. (Crystallographic information for these phases is summarized in the Appendix.) Note that the Al peak locations are given for a stoichiometry of ${\mathrm{Al}}_{0.924}{\mathrm{Mg}}_{0.076}$ to account for Mg in solution which causes the peaks to shift to lower $2\uptheta$ values owing to an increase in lattice parameter compared to a pure Aluminum lattice^52,53^. The insets in Fig. 6 highlight the peaks at $2\theta =31.241^\circ$ and $2\theta =32.408^\circ$ for the Al_13_CeMg_6_ ternary intermetallic phase. This phase is consistent with the Mg-rich regions observed by STEM-EDS (Fig. 5) and appears to be present in higher quantities for the as-fabricated near-eutectic alloy than for the hypereutectic alloy. Following HIP, these peaks decrease in intensity. The XRD data for the HIP specimens, particularly for the near-eutectic alloy, also show a shift of the FCC Al peaks to lower $2\uptheta$ values, consistent with an increase in lattice spacing, likely related here to an increase in the content of Mg in solution resulting from the dissolution of the Mg-rich Al_13_CeMg_6_ ternary compound.

### Mechanical test results

**Figure 7:**
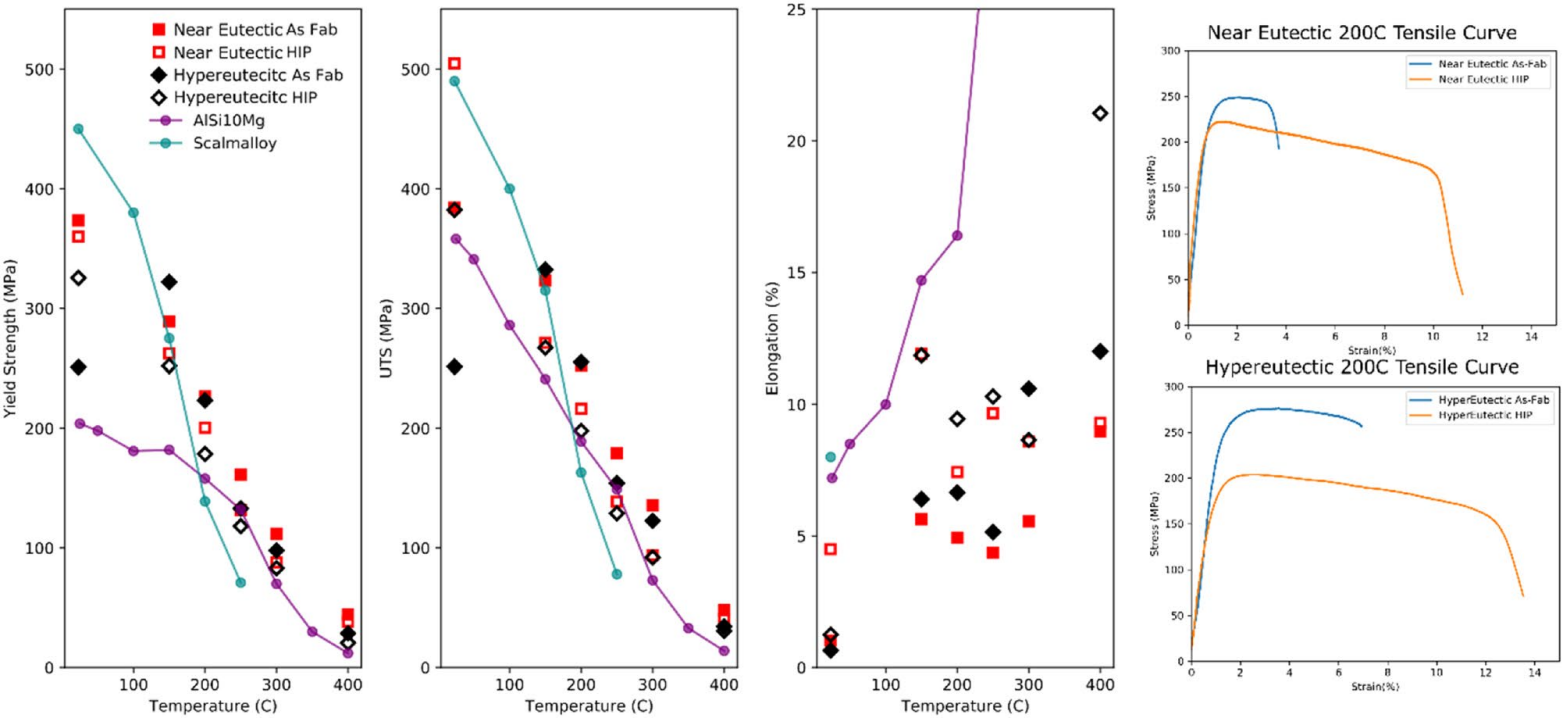
Averaged mechanical test data compared to some wrought alloys and published data for Scalmalloy^54^, and Al-10Si-Mg^16^.

Tensile properties for both alloys are shown in Fig. 7 as a function of temperature alongside representative tensile curves. For reference, the tensile properties are compared to additively manufactured Al-10Si-Mg^16^ and Scalmalloy^54^, a printable Al-Sc alloy. The near-eutectic alloy exhibits an average yield strength of 374 MPa and ultimate tensile strength of 384 MPa at room temperature in the as-fabricated condition. However, the average elongation at fracture in this condition is only about 1%. HIP of the near-eutectic alloy successfully increases the elongation to 4.5%, with only a small loss in yield strength at 360 MPa and, because the strength is no longer ductility limited, the average UTS increases to 505 MPa. With an average elongation of 0.65%, the hypereutectic alloy shows ductility limited behavior at room temperature, with yield and ultimate tensile strength of about 250 MPa. After HIP, the elongation improves slightly to 1.25%, resulting in an increase in the yield strength to 325 MPa and UTS to 382 MPa. The properties of the two alloys tend to converge with increasing temperature, with a characteristic reduction in strength and increase in elongation. Above 150 °C, the near-eutectic alloy tends to exhibit slightly higher strength, particularly in the as-fabricated condition, although, interestingly, the hyper-eutectic alloy shows higher elongation.

The yield strength of both alloys in this study exceed additively manufactured Al-10Si-Mg alloy at both room temperature and elevated temperatures, although this benefit is achieved with a corresponding reduction in room temperature ductility. Low temperature properties of additively manufactured Scalmalloy generally outperforms both alloys, but the Al–Ce–Mg alloys retain a higher fraction of their room temperature strength at elevated temperatures, and above 200 $^\circ \mathrm{C}$ out perform both common AM alloys in both the as-fabricated and HIP states.

**Figure 8:**
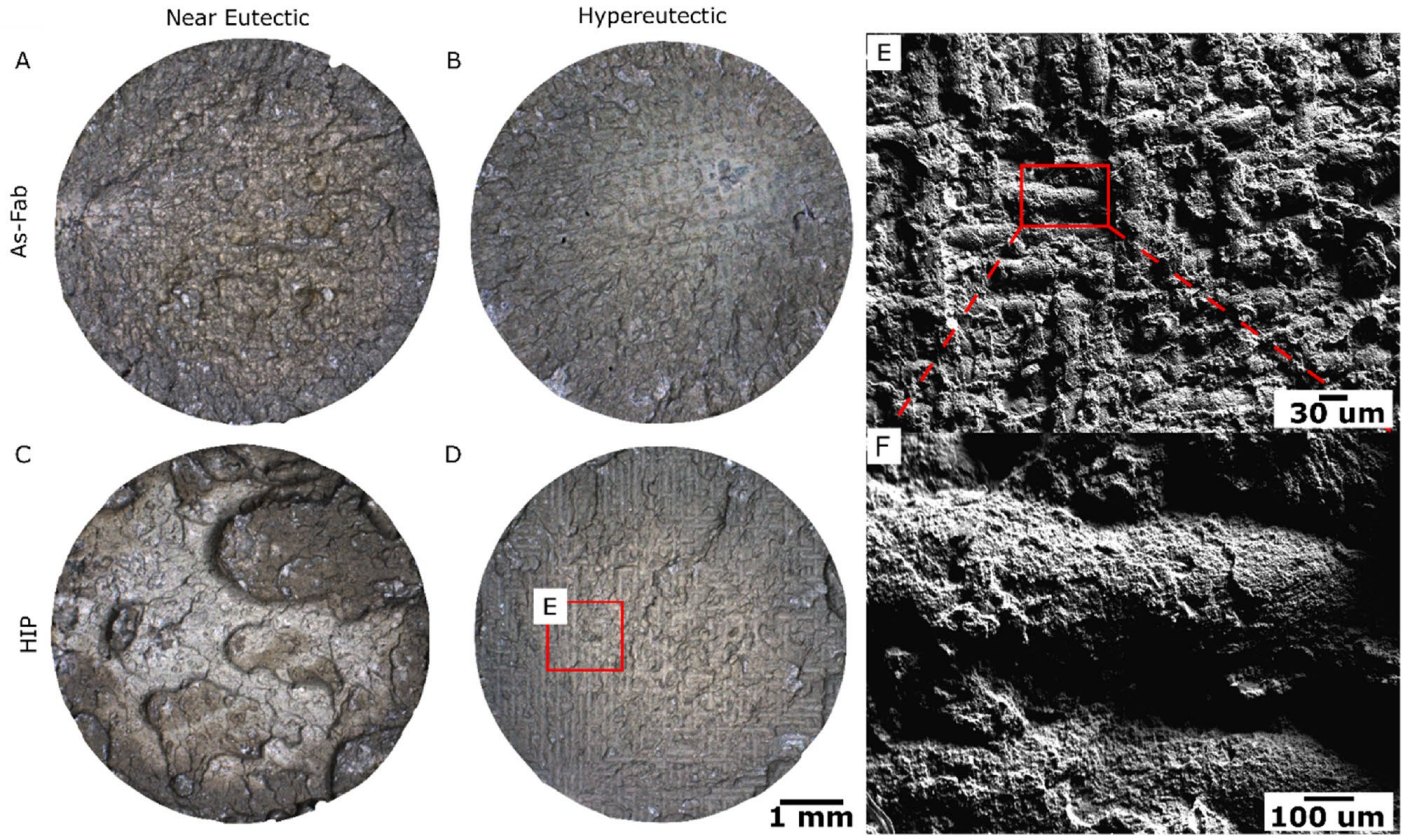
Fracture Surfaces of near-eutectic and hypereutectic alloys compared in the as-fabricated and HIP conditions. Where (**A**) and (**C**) are the Near Eutectic As-Fab and HIP fracture surfaces tested at $23^\circ C$. (**B**) and (**D**) are the Hypereutectic fracture surfaces at 23 $^\circ$ (**C**, **E**) An SEM image of the Hypereutectic HIP fracture surface showing failure along the weld pool edges, and (**F**) is a zoomed in region of (**E**).

Optical micrographs of the fracture surface of tensile specimens tested at room temperature are shown in Fig. 8. The fracture surface of the near-eutectic alloy is irregular in both the as-fabricated, Fig. 8A and HIP condition, Fig. 8C. However, in the hypereutectic alloy, particularly for the HIP condition, Fig. 8D, a repeating pattern that appears to have a spacing roughly equivalent to the hatch spacing of the scan pattern, averaging slightly around 0.11 mm. In Fig. 8E,F these patterns are investigated closer to show that it appears that failure occurs around weld pool boundaries in the microstructure, at room temperature. Similar patterns have been observed in the fracture surfaces of AM Al-Si alloys^17,55^, and in those cases, was attributed to the coarser microstructure observed at weld pool boundaries. Here, fracture appears to initiate from the primary Al_11_Ce_3_ intermetallic particles observed at the melt pool boundaries in the hyper-eutectic alloy (hyper-eutectic Zone 1 in Fig. 5). The particles tend to coarsen during HIP resulting in a fracture surface that appears to mimic the external weld pool boundaries.

## Discussion

### Scan pattern and porosity development

The large pores visible in the hyper-eutectic alloy sample that was fabricated using a conventional raster pattern (Fig. 3) are consistent with a keyhole mechanism for pore formation^48^. Keyholing is formed by the vaporization of molten metal causing a recoil pressure that depresses the surface of the liquid pool. Instabilities in the resulting vapor depression may result in the entrapment of the local atmosphere^48,56^. The Mg in the present alloys has a high vapor pressure and tends to preferentially vaporize under the high power density at the center of the laser beam, making these alloys prone to keyhole formation. This fact is consistent with the reduction in Mg in the printed parts relative to the powder (Table 1). To reduce keyhole formation, the skip raster technique was developed to increase spacing between sequential laser passes to distribute energy input more uniformly across the sample surface^42,57^. The reduction in surface temperature and corresponding vaporization is supported by the change in magnesium in the alloy after testing both scan strategies where the skip raster saw a full percent more magnesium retained in the part after production than a traditional raster.

Both alloys were processed using HIP to further reduce the porosity size. In the present alloys, a significant portion of their strength is derived from the fine intermetallic particle distribution formed during solidification. Coarsening of the Al_11_Ce_3_ particles under this condition was limited, and occurred primarily via diffusion along grain boundaries. The hypereutectic alloy had a greater degree of coarsening observed. Additional research is required to identify optimal HIP conditions that produce fully dense material without unnecessary microstructural coarsening.

### Phase formation and stability

**Figure 9:**
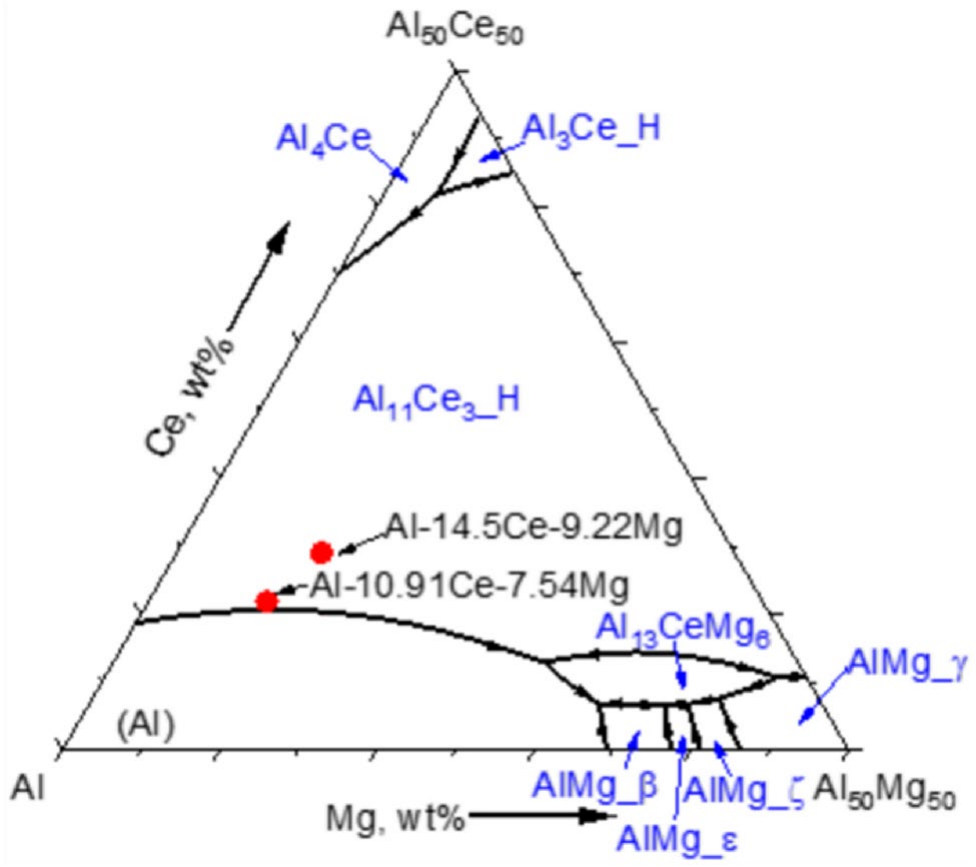
Calculated liquidus projection in the Al-rich region of the Al–Ce–Mg ternary system.

The calculated liquidus projection in the Al-rich region of the ternary Al–Ce–Mg system is shown in Fig. 9. The measured compositions of the near-eutectic and hypereutectic alloys are in the primary solidification region of ${\mathrm{Al}}_{11}{\mathrm{Ce}}_{3(\mathrm{H})}$, but the near-eutectic composition lies close to the binary Al + Al_11_Ce_3_ eutectic trough. The invariant reactions in this region are listed in the Appendix.

**Figure 10:**
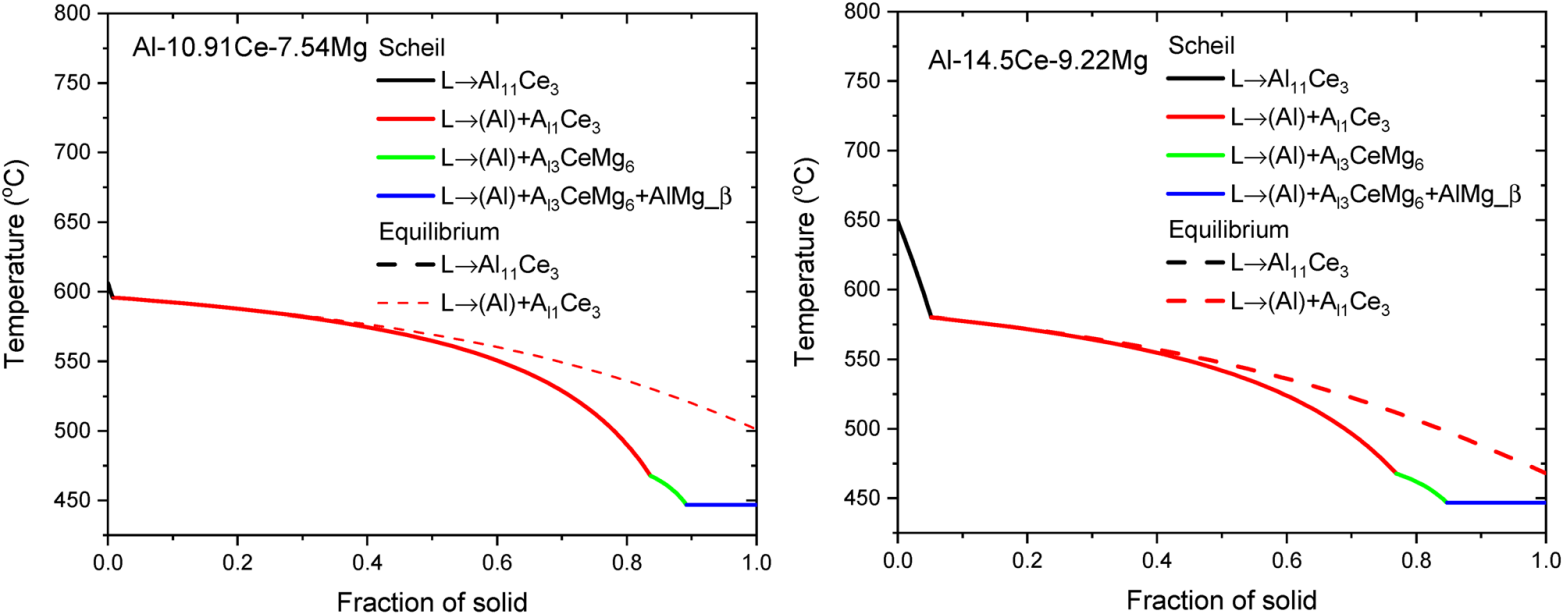
Scheil and Equilibrium Solidification Diagrams of Alloys Near Eutectic and Hypereutectic Respectively. The Calculation was done with the Chemical Composition of the powder.

The solidification paths of the measured compositions for the two alloys were calculated using two models: Scheil and Lever-rule model. Both the Scheil model and lever-rule models assume equilibrium at the solid–liquid interface. However, while the lever model assumes infinite diffusion in both solid and liquid, Scheil assumes no diffusion in the solid but complete mixing in the liquid. The resulting microsegregation profiles and predicted phases that form during solidification therefore represent extreme conditions that reasonably bound the behavior of most practical situations. The calculated solidification paths are plotted in Fig. 10, with dashed lines for lever rule and solid lines for Scheil model. The results clearly show that more phases are present in the Scheil model calculation due to increased microsegregation in this condition. The solidification temperature range is narrower in near-eutectic case. The set of phases predicted by the Scheil model are mostly consistent with those identified via XRD, although no β-AlMg was not observed in the as-fabricated AM samples.

It is notable that the Al_13_CeMg_6_ intermetallic compound is predicted for both alloys by the Scheil model, but not by the lever rule at equilibrium. This prediction is consistent with the Al_13_CeMg_6_ peaks found in the XRD spectra for the as fabricated samples, and the reduction in intensity of these peaks following HIP, suggesting that this phase is metastable in the solidification structure. In addition, as Al_13_CeMg_6_ dissolves during HIP, the Mg content is expected to move into solution in the FCC-Al matrix, tending to increase the lattice parameter. This effect explains the peak shift observed for Al in the XRD spectra.

### Solidification structure

A comparison of the solidification structure between the two alloys reveals interesting non-equilibrium behavior. As might be expected from its composition, the microstructure of the near-eutectic alloy consists of a eutectic structure of Al and Al_11_Ce_3_. This microstructure forms in two distinct morphologies: a globular structure near the edge of the melt pools, likely formed by partial re-melting of a previously formed microstructure, and a finer fibrous structure nearer the melt pool center. The hypereutectic alloy on the other hand forms a rich variety of structures: faceted primary Al_11_Ce_3_ particles in zone 1 near the melt pool boundary, followed by a fibrous Al + Al_11_Ce_3_ eutectic in zone 2, and, surprisingly, primary Al dendrites in zone 3.

**Figure 11:**
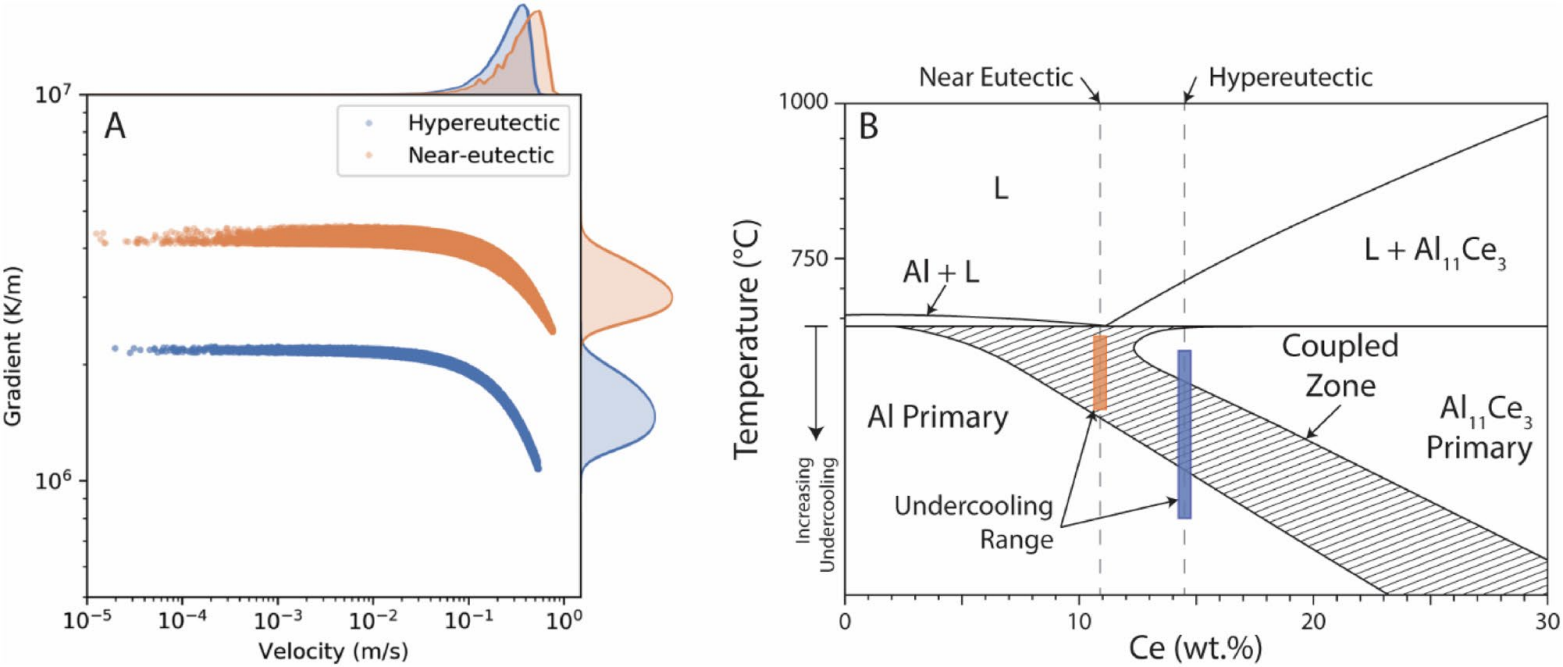
(**A**) Predicted distributions of the solid–liquid interface velocity and thermal gradient at the solidification front from the semi-analytical heat transfer model, showing Gaussian kernel density estimation of the statistical distributions, and (**B**) a schematic of the skewed coupled zone for the Al-Ce binary system, showing a wider required undercooling range to explain the observed microstructural variation in the hypereutectic alloy. Note that temperatures below the eutectic invariant reaction should be interpreted as increasing undercooling of the solid–liquid interface.

To rationalize these differences in microstructure evolution, the thermal characteristics of the process and the influence of composition on the non-equilibrium solidification structure must be considered. The semi-analytical heat conduction model (Sect. 2.8) was used to approximate the thermal conditions for the process conditions used in each alloy, including consideration for re-melting of subsequent layers. The solid–liquid interface velocity and the resultant thermal gradients were evaluated at the equilibrium eutectic temperature for direct comparison, and the resulting distributions are summarized in Fig. 11A. The heat transfer conditions are also spatially correlated with the melt pool geometry, with the highest gradient and lowest velocity at the melt pool boundary, and lowest gradient and highest velocity at the melt pool center.

The difference in solidification phase selection depends on the relative growth temperatures for the potential solidification modes^58^ as subject to the thermal conditions at the solid–liquid interface. Previous research on Al-Ce binary alloys has shown that non-equilibrium phase selection may occur depending on the local solidification conditions and result in patterning of difference solidification structures within a single melt pool^25^. In the Al-Ce system, the faceted nature of the Al_11_Ce_3_ phase suggests that its growth will be limited by high solid–liquid interfacial energy, which becomes increasingly dominant with increase solidification velocity. Primary solidification of Al_11_Ce_3_ is therefore easily suppressed at high solidification rates, explaining why it is not observed at all in the near-eutectic alloy, and only for low velocities at the melt pool boundaries in the hypereutectic alloy.

The differences in solid–liquid interfacial energy between Al dendrites and primary Al_11_Ce_3_ is also expected to lead to a skewed coupled eutectic zone (illustrated schematically in Fig. 11B for a hypothetical pseudo-binary Al-Ce system) characteristic of eutectic system feature one faced and one non-faceted phases^59–61^. In such systems, hypereutectic compositions may form eutectic structures or even primary dendrites of the hypoeutectic phase for large undercooling values. The appearance of Al dendrites in the hypereutectic alloy may be rationalized by considering such a system, which depends on the relative stability of the eutectic and Al dendrite growth modes. Based on the schematic representation in Fig. 11B, the difference in solidification structure between the two alloys may be understood if the larger range of undercooling for the hypereutectic alloy can be explained. We hypothesize that differences in constitutional supercooling play a significant role. The hypereutectic composition is richer in both Ce and Mg. For coupled eutectic growth of Al + Al_11_Ce_3_, both phases are lean in Mg, suggesting that the partitioning of Mg into the liquid will lead to a significant amount of constitutional undercooling that will tend to de-stabilize the eutectic growth relative to the binary system^62–64^. A higher amount of Mg in the hypereutectic alloy means that this source of undercooling will be greater than for the near-eutectic alloy. The growth of Al dendrites is therefore preferred under conditions where Mg concentration at the solid–liquid interface is significant. Coupled with the changes in thermal gradient, the differences in solidification modes may be rationalized. However, additional research will be required to quantify the influence of alloy chemistry and process characteristics on solidification mode selection, and improved understanding of the thermodynamic and kinetic properties of these alloys will also be required.

## Conclusion

This paper presented characterization of the microstructure and tensile properties for two Al–Ce–Mg alloys produced via additive manufacturing. One alloy was near-eutectic (Al–11Ce–7Mg) and the other hypereutectic (Al–15Ce–9Mg) with respect to the $\mathrm{L}\to \mathrm{Al}+{\mathrm{Al}}_{11}{\mathrm{Ce}}_{3}$ reaction. Mg was added as a solid solution strengthening element. However, preferential vaporization of Mg was observed, and the hypereutectic alloy was found to be prone to the formation of keyhole porosity. A custom skip raster scan pattern was successfully implemented to limit keyhole formation and a low-temperature HIP treatment was used to reduce porosity while limiting microstructural coarsening. The tensile properties of the two alloys were measured as a function of temperature in the as-fabricated and HIP conditions, and found to be superior in strength to common printable Al-Si alloys, although the room temperature ductility for both alloys was limited. Finally, the alloy microstructures were characterized through microscopy and x-ray diffraction. The microstructures were found to be the result of non-equilibrium solidification phenomena and highly dependent on both the heat transfer conditions during solidification and the differences in alloy chemistry.

## Supplementary Information

Supplementary Information
