# Supplementary material for: Microstructure and properties of additively manufactured Al–Ce–Mg alloys
Source: Sci Rep. 2021 Mar 26;11:6953. doi: 10.1038/s41598-021-86370-4 (PMC7998028; doi:10.1038/s41598-021-86370-4)
Supplement: Supplementary file 1 — Supplementary Information [file 41598_2021_86370_MOESM1_ESM.docx]

Microstructure and Properties of Additively Manufactured Al-Ce-Mg Alloys

K. Sisco^1^, A. Plotkowski^2^, Y. Yang^2^, D. Leonard^2^, B. Stump^2^, P. Nandwana^2^, R. R. Dehoff^3^, and S. S. Babu^4^

^1^ Material Science and Engineering, University of Tennessee-Knoxville, Knoxville, TN

^2^Materials Science and Technology Division, Oak Ridge National Laboratory, Oak Ridge, TN

^3^Manufacturing Science Division, Oak Ridge National Laboratory, Oak Ridge, TN

^4^Mechanical, Aerospace and Biomedical Engineering, University of Tennessee-Knoxville, Knoxville, TN

# APPENDIX A – Phases

The addition of cerium in the alloy is specifically to promote stable intermetallic phases at higher temperatures, specifically $Al_{11}Ce_{3}$ ^20,25,62^. The solubility of Cerium into aluminum is limited (.01 at %), forming a eutectic at 640$^{\circ}$C ^62^. The Magnesium in contrast has a high solubility in Aluminum (18.7%), and a eutectic forming around 450$^{\circ}$C ^50^. The magnesium is included as a solid solution strengthener to improve mechanical properties by substitution of aluminum atoms in the FCC matrix ^63–65^. The phase stability in the Al-Ce-Mg system is largely a breakdown of the two into Al-Ce and Al-Mg. This separation only sees overlap with the phase $Al_{13}\mathrm{CeM}g_{6}$ that is formed in an expected peritectic reaction between the liquid and $Al_{11}Ce_{3}$ for the Hypereutectic Alloy, and from the liquid in the Near Eutectic Alloy. This phase is expected to breakdown between 320$^{\circ}$C and 400$^{\circ}$C in a eutectoid reaction into $Al_{11}Ce_{3}$ and $\mathrm{AlM}g_{\beta}$ ^66^. The alloy design was selected around the Hypereutectic and hypereutectic to understand the difference in properties with the differences in expected solidification as seen the Scheil diagrams in the discussion.

Table 1: Lists the expected phases for both the Near Eutectic and Hypereutectic alloys. The beta AlMg phase is not included because it does not appear to be present in produced samples.

| **Phase** | **Space Group** | **Class** | **Lattice Parameters** | **References** |
| --- | --- | --- | --- | --- |
| Al | $\mathrm{Fm}\bar{3}m$ | Cubic | a = 4.05-4.09 Å | ^49,67,68^ |
| Al_11_Ce_3_ | Immm | Orthorhombic | a = 4.40 Å  b= 13.03 Å  c = 10.09 Å | ^69^ |
| Al_13_CeMg_6_ | P 63/mmc | Hexagonal | a = 5.25  c = 17.866 | ^66^ |

## APPENDIX B – Microstructure

In addition to the finely dispersed phase structure shown in Figure 3, the hypereutectic alloy as-fabricated microstructure was found to contain a distinct population of large Ce-rich intermetallic particles. These large particles are dispersed through the microstructure without a clear pattern. While the intermetallic particles in Figure 3 are generally below 1 µm, these larger particles seen in Figure 1, are 10-20 µm in size. Given that these particles are much larger than the rest of the solidification structure, we hypothesize that they were inherited from the original cast structure, present in the powder, and not completely dissolved during the AM process.


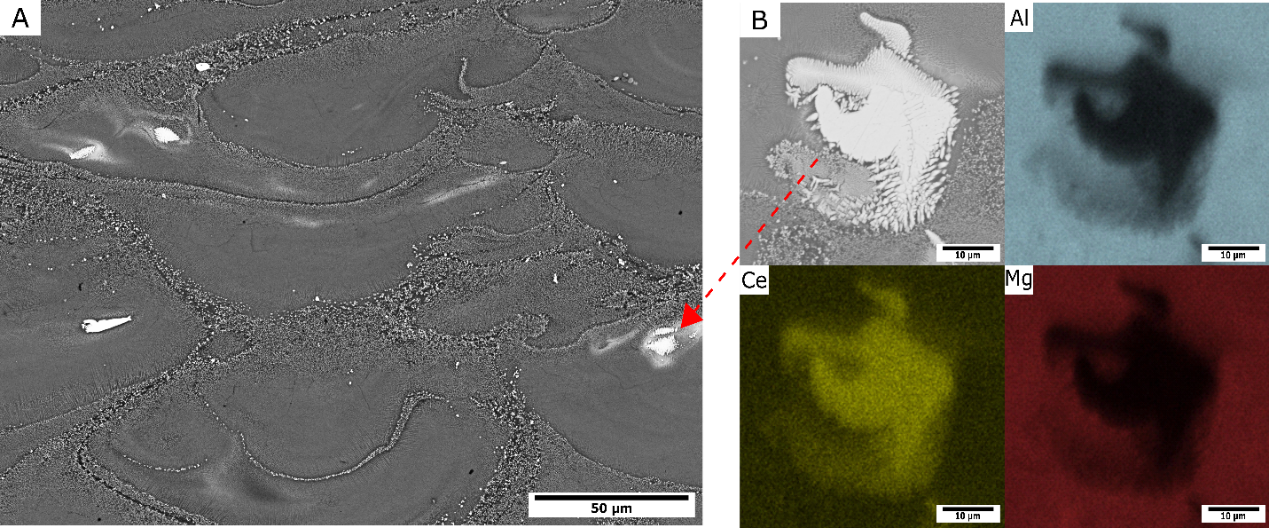


Figure 1: SEM micrograph and EDS maps of large Ce-rich particles found in the hypereutectic alloy.
